# Determining the cultural safety of chronic disease interventions for Aboriginal and Torres Strait Islander Australians: a scoping review

**DOI:** 10.3389/fpubh.2025.1462410

**Published:** 2025-01-23

**Authors:** Hannah Woodall, Sarah Larkins, Janani Pinidiyapathirage, Raelene Ward, Rebecca Evans

**Affiliations:** ^1^Research Office, Rural Medical Education Australia, Toowoomba, QLD, Australia; ^2^College of Medicine and Dentistry, James Cook University, Townsville, QLD, Australia; ^3^Rural Clinical School, Griffith University, Gold Coast, QLD, Australia; ^4^Future Drought Fund Hub (Research), University of Southern Queensland, Toowoomba, QLD, Australia

**Keywords:** cultural safety, chronic disease, Aboriginal and Torres Strait Islander health, scoping review, intervention

## Abstract

**Objectives:**

To assess how the cultural safety of primary care-based chronic disease interventions for Aboriginal and Torres Strait Islander Australians is determined.

**Methods:**

Scoping review of peer-reviewed evaluations of chronic disease interventions for Aboriginal and Torres Strait Islander patients, in which cultural safety is an outcome. Searches included Scopus, Informit, OVID Medline, Emcare and CINAHL including all articles published until September 2023.

**Results:**

Searches identified 2,225 articles. 1,854 articles underwent title and abstract screening, with 97 progressing to full text review. Twenty articles met the inclusion criteria. 75% (*n* = 15) of articles determined cultural safety based solely on Aboriginal and Torres Strait Islander peoples’ perspectives, with community acceptance as the most common means of determining cultural safety. In the analysed studies, elements contributing to cultural safety included practitioner behaviour (*n* = 15), knowledge (*n* = 6), skills (*n* = 1) and attitudes (*n* = 4), partnership with community (*n* = 4) and culturally safe services (*n* = 5), and graphics and artwork (*n* = 6). The inconsistent terminology and lack of definitions made comparison of studies challenging.

**Conclusion:**

This review underscores the importance of adopting the Australian Health Practitioner Regulation Agency (AHPRA) definition of cultural safety to standardise terminology and explore the many elements of cultural safety. It is recommended that cultural safety is defined by the community targeted by the intervention. Identification of elements of cultural safety will guide future interventions and reduce reliance on community acceptance as an indirect measure of cultural safety. If chronic diseases interventions are to effectively impact health equity, it is vital to understand cultural safety within these settings.

## Introduction

Chronic diseases are increasing in prevalence amidst a global shift towards non-communicable disease as a leading cause of morbidity and mortality (1). A chronic disease is any condition which has persisted, or is likely to persist for 6 months or longer (2). Within Australia, the 10 most common chronic diseases include arthritis, asthma, back conditions, cancer, chronic kidney disease, chronic obstructive pulmonary disease, diabetes mellitus, mental health conditions, osteoporosis and cardiovascular disease (3). Almost half of Australians experience at least one of these 10 conditions, with one fifth experiencing two or more (3). Chronic diseases contribute to 89% of Australian deaths and half of hospital admissions annually and thus cause significant health and financial burdens to individuals, communities, and health systems (3).

Chronic diseases also contribute to health inequity. The gap in life expectancy between Aboriginal and Torres Strait Islander and non-Indigenous Australians is well documented, and chronic diseases play a major role in this gap (4). Up to 70% of the increased burden of disease experienced by Aboriginal and Torres Strait Islander Australians is attributed to chronic disease (5). Ongoing health impacts of colonization and racism contribute to chronic disease prevalence and outcomes, making prevention and management an important target for intervention (5–7).

Australian chronic disease care primarily occurs in general practice, funded by Medicare, the Australian government-funded health insurance program (2, 8). Chronic disease interventions, supplementing GP or hospital management, have been trialed in many such settings (9). However, chronic disease interventions need to be culturally safe for Aboriginal and Torres Strait Islander patients, in order to reduce barriers to care and health inequities (10). Cultural safety is defined in Australia according to the Australian Health Practitioner Regulation Agency’s (AHPRA’s) Aboriginal and Torres Strait Islander Health Strategy group (11).

*“Cultural safety is determined by Aboriginal and Torres Strait Islander individuals, families and communities.*

*Culturally safe practise* (sic) *is the ongoing critical reflection of health practitioner knowledge, skills, attitudes, practising behaviours and power differentials in delivering safe, accessible and responsive healthcare free of racism.”* (11)

Determination of cultural safety is complicated by the diversity of terms and definitions in use. Many terms have been used in literature (e.g., cultural awareness, appropriateness, respect, security, humility, responsiveness) and the extent to which these overlap remains unclear (12–14). This paper uses the term cultural safety, in line with the AHPRA definition, but acknowledges the diversity of terms in use.

The need for culturally safe interventions requires that clinicians and researchers understand how cultural safety is facilitated and can be evaluated within the chronic disease context. This review will explore how cultural safety is currently evaluated to guide the incorporation of cultural safety into the design and evaluation of future interventions. Therefore, the aim of this review is to examine primary care-based chronic disease interventions targeting Aboriginal and Torres Strait Islander people. The review will evaluate how cultural safety has been evaluated within chronic disease interventions, and who determined these interventions to be culturally safe.

## Methods

### Search strategy

This review was conducted in line with the Joanna Briggs Institute (JBI) Manual for Evidence Synthesis (15). The review has been reported in accordance with the Preferred Reporting Items for Systematic reviews and Meta-Analyses extension for Scoping Reviews (PRISMA-ScR) checklist (16), which can be found in Supplementary Table 1. A scoping review methodology was chosen to allow exploration of current practices in determining cultural safety in chronic disease interventions within the heterogeneous research in this field (15).

Search terms were developed and tested based on expertise from the James Cook University library and previous literature. Chronic disease search terms were developed based on the 10 most common chronic diseases in Australia (3). Search terms are outlined in Supplementary Table 2. Search areas were combined with the AND operator. Five databases were included: Scopus, Informit, OVID Medline, Emcare and CINAHL, with searches adapted to database requirements. Searches were performed in September 2023 with Covidence™ used to identify and remove duplicates.

### Inclusion criteria

Articles were included if cultural safety as an outcome was included in evaluation of a chronic disease intervention. Given the diversity of terms used around cultural safety, the authors included any related term (e.g., cultural safety, sensitivity, appropriateness, awareness). Only Australian interventions were considered because of the potential differences in cultural safety between countries.

No year limit was placed on article inclusion. Only articles in English were included. Articles in pre-print were eligible for inclusion, but study protocols were excluded. Secondary research articles were not eligible for inclusion but were reserved for citation searching.

The search was limited to primary care-based interventions. Primary care settings included general practice, Aboriginal and Torres Strait Islander community-controlled health organizations (ACCHOs) or services equivalent to primary care (e.g., prison health services).

Only peer reviewed literature was searched. The screening process identified some grey literature which did not explicitly explore cultural safety. Further grey literature searching was not pursued since it was considered most important to explore determination of cultural safety in published evaluations.

### Article screening

Article screening was conducted using Covidence™. Screening was first conducted by title and abstract, followed by full text screening of remaining articles. Citation searching of all included articles as well as relevant secondary research articles was conducted. All articles were independently reviewed by two authors (A1 and A3), with differences resolved by discussion. Inclusion and exclusion criteria were the same across all phases of screening.

### Quality appraisal

Quality appraisal was included to facilitate consideration of the strength of the overall review findings. No articles were excluded based on quality appraisal. Quality appraisal was performed by the first author.

The Quality Assessment with Diverse Studies (QuADS) tool was used to assess methodological and reporting quality, allowing comparison across methodologies (17). Papers were scored from zero to three, with zero indicating no mention of the criteria and three indicating that the paper provided an explicit and detailed description (17).

The Aboriginal and Torres Strait Islander Quality Appraisal Tool (QAT) was used to explore research quality from an Aboriginal and Torres Strait Islander viewpoint (18). The 14 QAT criteria are scored as either “Unsure,” “Yes,” “No,” or “Partially” (18).

### Analysis

Data extraction was performed by the first author using Covidence™. Data extracted included: year of publication, location of intervention, nature of intervention, terminology and definitions used, who determined cultural safety and how cultural safety was determined.

Extracted data was exported to Excel™ and descriptive statistics performed to explore features of identified studies. Qualitative analysis using NVivo™ explored elements relevant to the development of cultural safety. Abductive coding was performed, informed by Braun and Clark’s methodology (19). Deductive coding explored features of cultural safety aligned with the AHPRA definition (11) with inductive development of data-driven codes to explore features beyond this definition.

## Results

**Figure 1:**
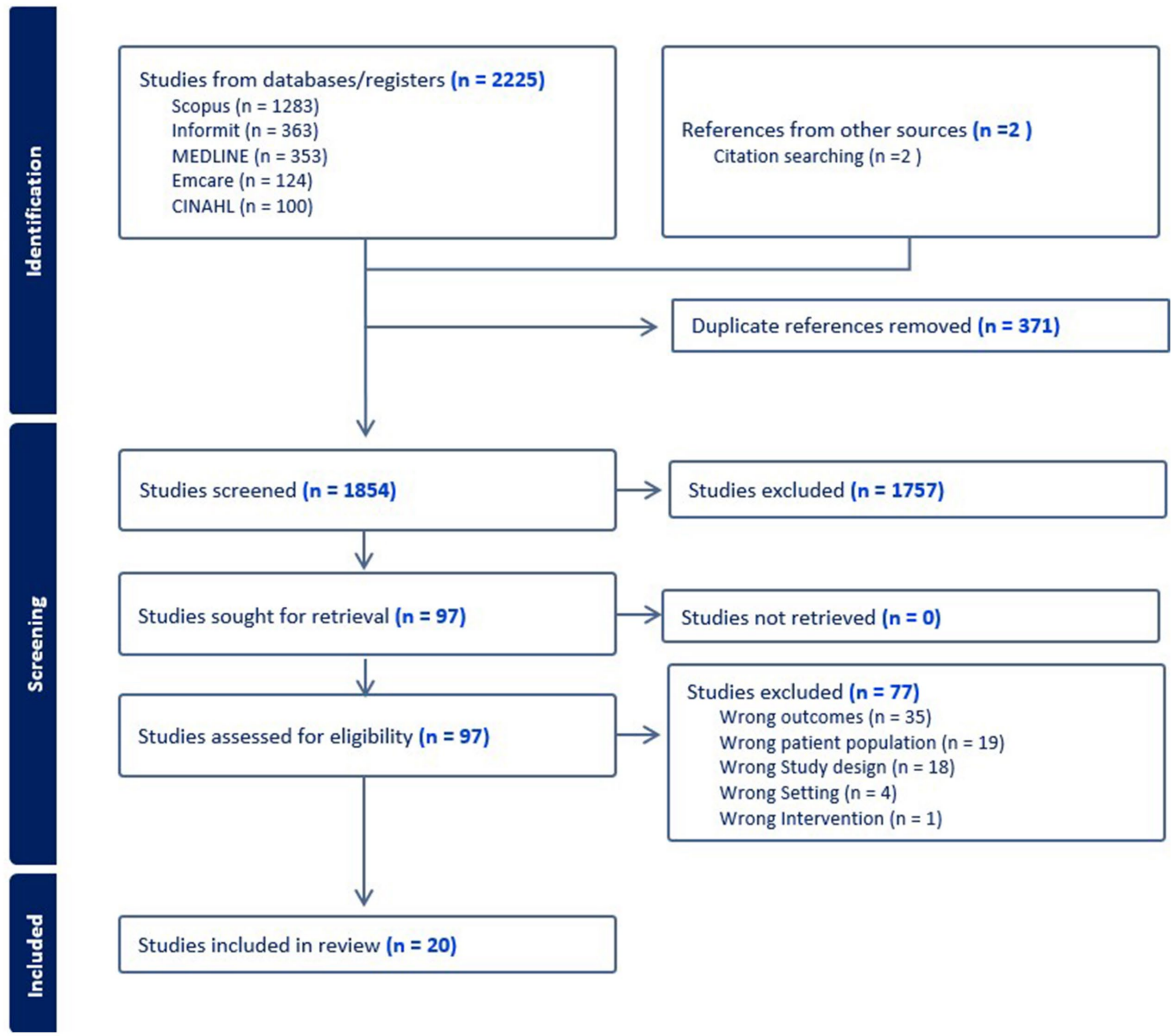
PRISMA flow diagram.

2,225 articles were identified through initial searches (Figure 1). Covidence™ identified and removed 371 duplicates, leaving 1,854 articles. Title and abstract screening excluded 1,757 studies. Full text screening excluded a further 79 articles, predominantly because cultural safety was not included as an outcome. Citation searching of identified articles and three relevant secondary research articles yielded an additional two articles for inclusion.

Twenty studies meeting the inclusion criteria were identified through the search. Studies were published between 2010 and 2023, with nine published since 2019. Nine studies (45%) evaluated a health program, with seven evaluating an educational activity or resource, and a further four evaluating a mobile application (Table 1). A quarter of studies were conducted in a metropolitan area, with a further quarter in remote areas as defined by the Modified Monash scale (20). The chronic conditions most targeted were Type 2 Diabetes (30% of studies) or mental health conditions (25%). Supplementary Table 3 contains a summary of included articles.

**Table 1:** Study characteristics of included articles.

Study characteristics			Number of studies (n)	Percentage of studies (%)
Study location	Metropolitan		5	25%
	Regional		3	15%
	Rural or remote		6	30%
	Mixed		4	20%
	Not reported/applicable		2	10%
Intervention type	Health Program or Activity		9	45%
	Educational activity or resource		7	35%
	Mobile application		4	20%
Disease targeted	Type 2 Diabetes Mellitus		6	30%
	Cardiovascular disease		3	15%
	Chronic respiratory disease		1	5%
	Chronic kidney disease		2	10%
	Mental health condition		5	25%
	Cancer		2	10%
	Other chronic disease		5	25%
Who determined cultural safety?	Aboriginal and Torres Strait Islander people only	Patients/community members	12	60%
		Aboriginal health professionals/facilitators	2	10%
		Patients/community members and Aboriginal health professionals/facilitators	1	5%
	Non-Indigenous people only	Researcher-determined based on criteria-based score	1	5%
	Both Aboriginal and Torres Strait Islander and non-Indigenous people	Both Aboriginal and Torres Strait Islander and Non-Indigenous health professionals/facilitators	1	5%
		Patients/community members and both Aboriginal and non-Indigenous health professionals/facilitators	2	10%
		Patients/community members and non-Indigenous health professionals/facilitators	1	5%

### Quality appraisal

Quality appraisal results are provided in Supplementary Table 3. Three papers consistently scored highly across the 13 QuADS criteria (21–23). Fourteen papers scored a 2 or 3 in over half of criteria (24–37) with only three papers scoring 0 or 1 in more than half of criteria (38–40).

A large proportion of the QAT criteria were not evident within included articles. The items most reported were those related to demonstrating community benefit and translation into sustainable change. The items which were least commonly reported were those related to intellectual or cultural property agreements, data control, a strengths-based approach and allowing opportunities for all team members to learn from one another.

### Terminology

Inconsistent terminology related to cultural safety was noted in these papers and included the following terms: culturally appropriate (*n* = 14), safe (*n* = 10), specific (*n* = 3), relevant (*n* = 3), aware (*n* = 2), acceptable (*n* = 2), sensitive (*n* = 1), informed (*n* = 1), responsive (*n* = 1), accessible (*n* = 1), capable (*n* = 1), adapted (*n* = 1), and secure (*n* = 1). Twelve papers used more than one term. Seven papers provided a definition for one or more terms used. One paper referenced the 2019 AHPRA definition (of seven articles published after 2020).

### Who determined cultural safety?

In 15 studies (75%), cultural safety was determined by Aboriginal and Torres Strait Islander people, with 12 basing this on the views of community and patients only (Table 1). In the remaining three papers, Aboriginal health professionals or program facilitators were also involved in determining cultural safety.

One study determined cultural safety based on a score administered by non-Indigenous researchers. This score was based on the DISCERN tool developed by the Cultural and Indigenous Research Centre and approved by an Aboriginal and Torres Strait Islander research team member (36, 41). The remaining four studies determined cultural safety based on the views of both Aboriginal and Torres Strait Islander and non-Indigenous representatives.

### How was cultural safety determined?

In half of the 20 studies, community acceptance was used as a proxy for cultural safety. Papers provided examples of positive feedback indicating satisfaction with the intervention or resource (21, 25, 26, 28–31, 34, 39, 40). In some studies, positive feedback was general and related to the program overall (40) whereas others received more specific feedback (e.g., traditional language or yarning) (29, 34). Nine studies determined cultural safety based on either specific questions about cultural safety (24, 27, 35, 38) or a combination of community acceptance and specific questions (22, 23, 32, 33, 37).

### What elements contributed to cultural safety?

Three papers did not explore which elements contributed to cultural safety of the intervention (28, 37, 38). Amongst the remaining papers, identified elements aligned with the AHPRA cultural safety definition.

Supplementary Table 4 summarises cultural safety elements identified within the included papers. Practicing behaviours and practitioner knowledge were the two elements of the AHPRA definition most frequently identified by papers. Practising behaviours included providing holistic care, allowing time for silence, narrative based approaches, yarning circles, and involvement of Aboriginal health workers (21, 22, 24, 25, 31, 34). In educational resources or mobile application interventions, practising behaviours related to ensuring language was understandable and incorporating traditional language (29, 30, 32). Knowledge elements predominantly related to being aware of the impact of colonization and subsequent trauma on the health of Aboriginal and Torres Strait Islander people (22, 30, 36).

Other elements of the AHPRA definition were less comprehensively explored. References to attitudes related to avoiding judgement and making the intervention a safe space (22, 24, 31, 33). Power differentials were discussed only in relation to a client’s level of control within the program (34, 40). Power differentials between health professionals or facilitators and participants were not explicitly discussed. Only one paper referred to critical reflection and the impact of the health professionals’ own culture (22). This same paper was the only one to reference health professional skills and the AHPRA definition (22). The importance of interventions being free of racism was not explicitly discussed in any papers. One paper referred to avoiding stereotypes about Aboriginal and Torres Strait Islander people (36).

Three additional elements were identified beyond the AHPRA definition (Supplementary Table 4). The role of community in developing and supporting interventions was highlighted (26, 30, 36), along with the importance of partnering with existing culturally safe services (21, 31, 39). Several papers discussed the perceived importance of graphics and artwork for cultural safety in educational materials or mobile applications (23, 33, 36). In one face to face intervention, health professionals reported the importance of flags and artwork, although it is not reported whether these individuals identified as Aboriginal and Torres Strait Islander (22).

## Discussion

This review sought to explore interventions evaluated as culturally safe in a primary care setting for Australian Aboriginal and Torres Strait Islander people, to examine who determined the interventions to be culturally safe and how this determination was made.

### What is cultural safety?

The definition of cultural safety remains a point of contention. Only one of seven studies published after 2020 referenced the 2019 AHPRA definition. A broad variety of terms are used in the included papers. Some authors may use these terms interchangeably, while others may seem them as distinct or a progression in competence (12, 42). The variety of terms used, often without definition, complicates the literature in this field. Based on these findings, it is recommended that Australian studies use the accepted AHPRA definition, developed through community consultation, to provide consistency (11). Where alternative terms are used, we recommend that these terms are clearly defined to provide clarity.

### Who determined cultural safety?

In three quarters of identified studies, cultural safety was defined by Aboriginal and Torres Strait Islander people only. Four studies determined cultural safety based on both Aboriginal and Torres Strait Islander and non-Indigenous individuals. In these studies, it was often difficult to determine how many participants were Aboriginal or Torres Strait Islander, and which participants determined cultural safety. As a result, it is recommended that future studies clearly report who was responsible for determining cultural safety.

### How was cultural safety determined?

Ten studies used community acceptance as a proxy measure for cultural safety, the validity of which is unclear. Community acceptance may indicate a community’s decision that an intervention is culturally safe. Conversely, community acceptance may indicate a lack of alternative options (particularly in rural and remote areas) or may reflect that an individual enjoyed the program without considering if it was culturally safe for the wider community. Without specifically questioning cultural safety, it is difficult to be certain whether interventions are considered culturally safe.

Nine studies employed specific questions about cultural safety either alone or in addition to community acceptance measures. However, these studies did not seek to understand the patient’s definition of terms used (27). Thus, given the variety of terms and definitions in use, the interpretation of these questions remains challenging.

One article used a score to determine cultural safety. This score was based on the DISCERN criteria, accepted by an Aboriginal and Torres Strait Islander team member (36, 41). The use of this tool is limited to health education materials, but requires further validation in this context.

Thus, determination of the cultural safety of interventions remains challenging. At this stage, the tools predominantly in use are the presence of community acceptance and/or specific questions about cultural safety. In the absence of clear tools to measure culture safety, it is recommended that specific questions are asked, alongside an exploration of participants’ definition and understanding of cultural safety.

### Elements of cultural safety identified

Seventeen papers described elements contributing to cultural safety, many of which aligned with elements identified within the AHPRA definition of cultural safety. The most common elements identified related to practicing behaviors and practitioner knowledge. These elements provide insight into the behaviors and knowledge which may contribute to the cultural safety of future interventions (e.g., holistic care, narrative approaches, traditional language, understanding the impact of colonization).

Elements within the AHPRA definition which were not frequently covered also provide insights about ways to improve evaluations. The only paper that considered critical reflection and health professional skills was the one to have utilized the AHPRA definition. Thus, wider use of this definition may prompt more studies to consider cultural safety in greater detail.

The lack of consideration of power differentials also impacts determination of cultural safety, particularly when evaluations were performed by intervention staff. While some studies reported relationships between the evaluating team and the participants and intervention (22, 24), many others omitted this information. Clear reporting of such relationships is vital to appropriately considering power differentials and their potential influence in determining cultural safety.

No interventions explicitly discussed being “free of racism,” other than one paper discussing avoidance of stereotypes. The lack of racism may be presumed. However, given the prevalence of racism within Australia (43), greater exploration of racism within interventions is important.

Finally, some interventions identified elements of cultural safety beyond the AHPRA definition. Papers recognised the importance of community in developing and supporting interventions, to ensure that the developed intervention meets community needs and is culturally safe in its context. Some interventions noted that links with existing services (such as ACCHOs) which were known to be culturally safe, improved the cultural safety of the intervention. Such linkages provided a safe space for the intervention and were felt to confer cultural safety.

As a result, it is recommended that future evaluations use the elements of the AHPRA definition to guide cultural safety evaluations; considering knowledge, behaviour, skills, reflection, power differentials and racism. In addition, the importance of partnering with existing culturally safe services and community in developing and evaluating interventions cannot be understated.

## Implications

The findings of this study have implications for the design of future chronic disease interventions, and the evaluation of existing ones. It is recommended that cultural safety be explicitly explored in intervention design and evaluation, avoiding the use of proxy measures such as community acceptance. The cultural safety of interventions should be determined by Aboriginal and Torres Strait Islander individuals and communities, exploring what cultural safety means to those involved. Cultural safety evaluations are also recommended to report who determined cultural safety and what definition/s were used in making these assessments.

Finally, this review suggests that the AHPRA definition can be used to guide cultural safety evaluations, considering each element of the definition. These results also highlights the importance and benefits of partnering with community and culturally safe organisations in co-designing chronic disease interventions.

### Areas for future study

Further study is required, based on consistent terminology and definitions, to explore the relationship between the AHPRA definition and cultural safety of interventions, and identify additional elements which may be required. Further study within other countries would be valuable to add to the literature around culturally safe chronic disease interventions globally.

## Limitations

Our review was limited to interventions conducted in a primary care setting within Australia and may not be generalizable to interventions based in hospitals or community settings or outside of Australia. The exclusion of study protocols and grey literature may also have limited the consideration of smaller or more recent interventions. As is accepted practice in scoping reviews, no protocol was prospectively registered.

Quality appraisal was conducted to guide interpretation of the studies, but studies were not excluded based this appraisal. Quality appraisal indicated limited reporting especially for the QAT, impacting interpretation of included studies. Given the importance of ensuring cultural safety is determined by Aboriginal and Torres Strait Islander community, it is also important that research is assessed from this perspective. The QAT, which was published in 2022 (after 18 of the included papers) provides a useful tool for this purpose. It is hoped that greater recognition of this tool will improve reporting in future studies.

## Conclusion

Chronic disease interventions are important in supporting Aboriginal and Torres Strait Islander people with chronic disease. However, it is vital to understand how to determine the cultural safety of these services. Future studies should strive to ensure that cultural safety is determined by the community for whom the intervention is intended (rather than non-Indigenous individuals or program facilitators). The use of the existing AHPRA definition of cultural safety is recommended to ensure consistency of terms, and to encourage the exploration of the many aspects of cultural safety. Definition of such elements will reduce reliance on community acceptance as a proxy measure for cultural safety. Improving the evaluation of cultural safety within chronic disease interventions is one step towards improving access to these services and working towards health equity.
