# Supplementary material for: Determining the cultural safety of chronic disease interventions for Aboriginal and Torres Strait Islander Australians: a scoping review
Source: Front Public Health. 2025 Jan 23;13:1462410. doi: 10.3389/fpubh.2025.1462410 (PMC11799238; doi:10.3389/fpubh.2025.1462410)
Supplement: Supplementary file 1 [file Table_1.DOCX]

**Supplementary Materials:**

Table 1: Preferred Reporting Items for Systematic reviews and Meta-Analyses extension for Scoping Reviews (PRISMA-ScR) Checklist

| **SECTION** | **ITEM** | **PRISMA-ScR CHECKLIST ITEM** | **REPORTED ON PAGE #** |
| --- | --- | --- | --- |
| **TITLE** | | | |
| Title | 1 | Identify the report as a scoping review. | 1 |
| **ABSTRACT** | | | |
| Structured summary | 2 | Provide a structured summary that includes (as applicable): background, objectives, eligibility criteria, sources of evidence, charting methods, results, and conclusions that relate to the review questions and objectives. | 2 |
| **INTRODUCTION** | | | |
| Rationale | 3 | Describe the rationale for the review in the context of what is already known. Explain why the review questions/objectives lend themselves to a scoping review approach. | 3-4 |
| Objectives | 4 | Provide an explicit statement of the questions and objectives being addressed with reference to their key elements (e.g., population or participants, concepts, and context) or other relevant key elements used to conceptualize the review questions and/or objectives. | 4 |
| **METHODS** | | | |
| Protocol and registration | 5 | Indicate whether a review protocol exists; state if and where it can be accessed (e.g., a Web address); and if available, provide registration information, including the registration number. | N/A |
| Eligibility criteria | 6 | Specify characteristics of the sources of evidence used as eligibility criteria (e.g., years considered, language, and publication status), and provide a rationale. | 4 |
| Information sources* | 7 | Describe all information sources in the search (e.g., databases with dates of coverage and contact with authors to identify additional sources), as well as the date the most recent search was executed. | 4 |
| Search | 8 | Present the full electronic search strategy for at least 1 database, including any limits used, such that it could be repeated. | Supplementary Table 2 |
| Selection of sources of evidence† | 9 | State the process for selecting sources of evidence (i.e., screening and eligibility) included in the scoping review. | 4 |
| Data charting process‡ | 10 | Describe the methods of charting data from the included sources of evidence (e.g., calibrated forms or forms that have been tested by the team before their use, and whether data charting was done independently or in duplicate) and any processes for obtaining and confirming data from investigators. | 5 |
| Data items | 11 | List and define all variables for which data were sought and any assumptions and simplifications made. | 4-5 |
| Critical appraisal of individual sources of evidence§ | 12 | If done, provide a rationale for conducting a critical appraisal of included sources of evidence; describe the methods used and how this information was used in any data synthesis (if appropriate). | 5 |
| Synthesis of results | 13 | Describe the methods of handling and summarizing the data that were charted. | 4-5 |
| **RESULTS** | | | |
| Selection of sources of evidence | 14 | Give numbers of sources of evidence screened, assessed for eligibility, and included in the review, with reasons for exclusions at each stage, ideally using a flow diagram. | 5 |
| Characteristics of sources of evidence | 15 | For each source of evidence, present characteristics for which data were charted and provide the citations. | 5, Table 1, |
| Critical appraisal within sources of evidence | 16 | If done, present data on critical appraisal of included sources of evidence (see item 12). | 5-6 |
| Results of individual sources of evidence | 17 | For each included source of evidence, present the relevant data that were charted that relate to the review questions and objectives. | 5-7 and Supplementary Table 4 |
| Synthesis of results | 18 | Summarize and/or present the charting results as they relate to the review questions and objectives. | 5-7 and Supplementary Table 4 |
| **DISCUSSION** | | | |
| Summary of evidence | 19 | Summarize the main results (including an overview of concepts, themes, and types of evidence available), link to the review questions and objectives, and consider the relevance to key groups. | 7-9 |
| Limitations | 20 | Discuss the limitations of the scoping review process. | 9 |
| Conclusions | 21 | Provide a general interpretation of the results with respect to the review questions and objectives, as well as potential implications and/or next steps. | 10 |
| **FUNDING** | | | |
| Funding | 22 | Describe sources of funding for the included sources of evidence, as well as sources of funding for the scoping review. Describe the role of the funders of the scoping review. | 10 |

JBI = Joanna Briggs Institute; PRISMA-ScR = Preferred Reporting Items for Systematic reviews and Meta-Analyses extension for Scoping Reviews.

* Where *sources of evidence* (see second footnote) are compiled from, such as bibliographic databases, social media platforms, and Web sites.

† A more inclusive/heterogeneous term used to account for the different types of evidence or data sources (e.g., quantitative and/or qualitative research, expert opinion, and policy documents) that may be eligible in a scoping review as opposed to only studies. This is not to be confused with *information sources* (see first footnote).

‡ The frameworks by Arksey and O’Malley (6) and Levac and colleagues (7) and the JBI guidance (4, 5) refer to the process of data extraction in a scoping review as data charting*.*

§ The process of systematically examining research evidence to assess its validity, results, and relevance before using it to inform a decision. This term is used for items 12 and 19 instead of "risk of bias" (which is more applicable to systematic reviews of interventions) to include and acknowledge the various sources of evidence that may be used in a scoping review (e.g., quantitative and/or qualitative research, expert opinion, and policy document).

*From:* Tricco AC, Lillie E, Zarin W, O'Brien KK, Colquhoun H, Levac D, et al. PRISMA Extension for Scoping Reviews (PRISMAScR): Checklist and Explanation. Ann Intern Med. 2018;169:467–473. [doi: 10.7326/M18-0850](http://annals.org/aim/fullarticle/2700389/prisma-extension-scoping-reviews-prisma-scr-checklist-explanation).

*Table 2: Search Terms*

| Search area | Terms used |
| --- | --- |
| Aboriginal and Torres Strait Islander | aborig* OR torres OR australoid OR indigenous OR "oceanic ancestry group" OR oceanian* OR (pacific AND island* ) OR (native AND hawaiian* ) OR ( first AND nation* ) |
| Cultural safety / sensitivity / awareness etc | Cultur* |
| Chronic disease | (Chronic AND disease*) OR (Chronic* AND ill*) OR diabet* OR cardiac OR heart OR cardio* OR kidney OR renal OR nephropathy OR arthriti* OR oligoarthriti* OR polyarthriti* OR osteoarthritis OR (back and ache*) OR coccydynia OR (back AND pain*) OR vertebrogenic OR (chronic AND pain) OR dorsalgia OR asthma* OR malignan* OR tumor* OR tumour* OR neoplas* OR copd OR coad OR "chronic obstructive lung" OR "chronic obstructive pulmonary" or "chronic airflow" OR mental* OR psychiatric OR insanity OR psychic dis* OR psychopatholog* OR osteoporos* OR decalcification |

Table 3: Alignment of elements of cultural safety with the AHPRA definition

| **Elements of cultural safety of an intervention** | | **Examples from included studies** |
| --- | --- | --- |
| Elements of the AHPRA definition | Critical reflection | “Health professionals said they needed to recognise their own culture and the culture of the patients.” (21) |
|  | Knowledge | “Health professionals’ knowledge, skills and confidence in working with Aboriginal people improved following attendance at the cultural awareness training and involvement in the program.” (21) (Cultural awareness program for health professionals incorporated into program)  “Historical factors were seen to cause mental ill health and influence acceptability of the apps to participants. The negative impact of colonization on the well-being of Aboriginal and Torres Strait Islander people was highlighted in the discussion, along with uncertainty about the role of apps in addressing such concerns” (29)  “A brochure produced by the Australian Government Department of Health and The University of Sydney Matilda Centre was the only resource that acknowledged intergenerational trauma as one of the underlying causes of drugs misuse. On the other hand, another resource stated that alcohol use may contribute to separation from culture. “ (35) |
|  | Attitudes | “The look and feel” of the app and its original multimedia creations were appreciated as culturally relevant and “right.” This created a safe space for Indigenous people to explore mental health coping strategies without the shame factor” (32)  “Participants felt that the health service environment was comfortable and safe, where they were not judged, and lessened feelings of guilt connected with previous experiences within the mainstream healthcare system.” (21) |
|  | Practising behaviours | “Being interested in them and their lives, providing holistic care and removing many of the everyday stressors and worries associated with living with complex chronic diseases for them and their family members.” (20)  “Health professionals said they needed to recognise their own culture and the culture of the patients, including allowing time for silence. They also found that analogies worked well, particularly connecting with the land.” (21)  “So, cultural safety (which includes use of Aboriginal facilitators, both ways knowledge sharing, respect for culture and elders, and local language use) produces an environment conducive to mutual support, reduced stigma and a safe space to talk about suicide.” (23)  “About one-third of participants commented on how the language in MBOT was familiar and understandable: “The information was understandable and it made sense, and the language used was clear. “” (31)  “A cultural consideration was the use of a narrative/story-based approach,” (31)  “Clear, concise, and relevant language were acknowledged as important. Words that could be difficult to understand needed to be supported by explanations or short video clips” (29)  “The formative research suggested that promoting the GHS should use straightforward language and strong Aboriginal visuals and colours; emphasise that it is a free confidential service, working closely with Aboriginal Community Controlled Health Services (ACCHS) and be sensitive to Aboriginal needs by providing a personalised service. “ (30)  “careful integration of skilled Aboriginal health workers to encourage participation and clearly communicate health information. Community members valued this component of the project and in some cases this was contrasted with the poor communication in mainstream health services – a finding mirrored in previous research.25,26 It seems likely that sustained integration of Aboriginal health workers and community in an ongoing program would benefit the understandings needed for better prevention and management of kidney disease.” (24) “It was concluded that developing resources in Aboriginal languages is important to Aboriginal people as it helps validate their cultures, enhances engagement and provides opportunity to develop health vocabularies where there is no existing translatable term.”“ (28)  “Design aspects of the clinic model, such as yarning circles, group appointments, and education sessions, were identified by participants as making them feel more likely to attend and encouraged Community engagement. “ (33) |
|  | Skills | “Participants felt that the health service environment was comfortable and safe, where they were not judged, and lessened feelings of guilt connected with previous experiences within the mainstream healthcare system. Health professionals’ knowledge, skills and confidence in working with Aboriginal people improved following attendance at the cultural awareness training and involvement in the program.” (21) |
|  | Power differentials | “This pilot program began through an invitation from within an Aboriginal and Torres Strait Islander community-controlled health organisation, which ensured the student was sent where there was a stated community need and desire for music therapy. Goals for the pilot program were developed directly in consultation with clients to ensure they were culturally valued and not lead by the student's own cultural values” (39)  “Interestingly, our study findings also demonstrated a positive response by Community members to the integration of undergraduate podiatry students into the clinical service delivery model. The strong positive feedback on this element of the Buridja Clinic was noteworthy, with this seen by participants as both an opportunity for them to generate change in perception of Aboriginal and Torres Strait Islander Peoples and health outcomes, and part of their own role in the clinic. This may have contributed to an increased sense of ownership of, and engagement with, the clinic, which is essential for success. “ (33) |
|  | Free of racism | “avoided stereotypes” (35) |
| Additional elements | Role of community in developing culturally safe programs | “Our participants identified community involvement in development as a good strategy for improving acceptability, adherence, and uptake of e-mental health apps in a location specific community.” (29)  “By working in collaboration with HF *[heart failure]* experts, Aboriginal researchers and patients, a culturally safe HF resource has been developed for Aboriginal and Torres Strait Islander patients.” (25)  “Developed in collaboration with local community” (criteria for cultural safety) (35) |
|  | Linkage with existing culturally safe services | “All believed that it was a natural extension of the primary health care service, and reflected the health service’s aims and vision of providing high quality, accessible, culturally appropriate care for Aboriginal and Torres Strait Islander peoples.” (20)  “Being based at a community-controlled AMS was seen as critical, as it linked the client in their home to the AMS and to the hospital and provided continuity between these services.” (30)  “The venue was acceptable, and the female facilitator felt the DAHS venue contributed to cultural safety.” (38) |
|  | Graphics and artwork | “Twenty- seven out of 30 resources included culturally relevant artwork or images” (35)  “Participants liked how colorful it was and the use of cultural imagery and stories “ (22)  “Some health professionals felt that a culturally safe space was provided by introducing Aboriginal and Torres Strait Islander flags and artwork.“ (21)  ““The look and feel” of the app and its original multimedia creations were appreciated as culturally relevant and “right.”” (32) |

**Table 4: Summary of included articles**

| **Year, author** | **Title** | **Location*** | **Disease targeted, Intervention type** | **Terminology used** | **Definition included (Yes / No/) †** | **Who determined cultural safety?** | **How was cultural safety determined?** | **Quality assessment with diverse studies tool scores‡** | | | | **Aboriginal and Torres Strait Islander QAT ^**  (Y= yes, N= no, P= partially, U= unclear) | |
| --- | --- | --- | --- | --- | --- | --- | --- | --- | --- | --- | --- | --- | --- |
| 2023  Amanda et al (35) | Evaluation of the usability, content, readability and cultural appropriateness of online alcohol and other drugs resources for Aboriginal and Torres Strait Islander Peoples in New South Wales, Australia | NSW, rurality not reported | Alcohol and other drug use, Online educational resources | Culturally appropriate | No | Researcher based on a criteria | A score based on the DISCERN instrument (40) | C1 = 2 C2 = 3  C3 = 3 C4 = 2  C5 = 0 C6 = 3  C7 = 1 | C8 = 1  C9 = 1  C10 = 2  C11 = 1  C12 = 0 C13 = 2 | | | Q1 = U  Q2 = U  Q3 = U  Q4 = P  Q5 = U  Q6 = U  Q7 = U | Q8 = U  Q9 = U  Q10 = U  Q11 = P  Q12 = P  Q13 = U  Q14 = U |
| 2016 Askew et al (20) | Investigating the feasibility, acceptability and appropriateness of outreach case management in an urban Aboriginal and Torres Strait Islander primary health care service: a mixed methods exploratory study | QLD, metropolitan | Type 2 Diabetes, cardiovascular disease, chronic respiratory or kidney disease,  At-home case management | Culturally appropriate | No | Patients / community members | Community acceptance | C1 = 3 C2 = 3  C3 = 3 C4 = 2  C5 = 2 C6 = 2  C7 = 3 | C8 = 3  C9 = 3 C10 = 3 C11 = 3 C12 = 2 C13 = 3 | | | Q1 = U Q2 = Y  Q3 = U Q4 = P  Q5 = U Q6 = U  Q7 = U | Q8 = U Q9 = U Q10 = U  Q11 = Y Q12 = Y  Q13 = U Q14 = U |
| 2017 Bierbaum et al (26) | Challenges to uptake of cancer education resources by rural Aboriginal Health Workers: the Cancer Healing Messages flipchart experience. | SA, mixed rurality | Cancer, educational resource | Culturally appropriate | No | Aboriginal health professional/ facilitator | Specific questions about cultural safety | C1 = 2 C2 = 3  C3 = 3 C4 = 2  C5 = 1 C6 = 2  C7 = 2 | C8 = 3  C9 = 3 C10 = 1  C11 = 2  C12 = 0  C13 = 2 | | | Q1 = U Q2 = U  Q3 = U Q4 = U  Q5 = U Q6 = U  Q7 = U | Q8 = U Q9 = U Q10 = U  Q11 = P Q12 = P  Q13 = U Q14 = U |
| 2014 Browne et al (36) | Feltman: evaluating the acceptability of a diabetes education tool for Aboriginal health workers | VIC, rurality not reported | Diabetes, educational resource | Culturally appropriate  Culturally relevant | No | Aboriginal and non-Aboriginal health professional/ facilitator | Community acceptance; Specific questions about cultural safety | C1 = 2 C2 = 2  C3 = 2 C4 = 2  C5 = 2 C6 = 0  C7 = 2 | | C8 = 1 C9 = 1 C10 = 0  C11 = 2  C12 = 0  C13 = 2 | | Q1 = U Q2 = U  Q3 = U Q4 = U  Q5 = U Q6 = U  Q7 = U | Q8 = U Q9 = U Q10 = U  Q11 = P Q12 = Y  Q13 = U Q14 = U |
| 2015 Clark et al (25) | Development and feasibility testing of an education program to improve knowledge and self-care among Aboriginal and Torres Strait Islander patients with heart failure. | QLD, metropolitan | Heart failure, educational resource | Cultural safety | Yes | Patients and community members | Community acceptance | C1 = 2 C2 = 3  C3 = 3 C4 = 2  C5 = 0 C6 = 1  C7 = 2 | | | C8 = 3 C9 = 3 C10 = 1  C11 = 3  C12 = 2  C13 = 2 | Q1 = U Q2 = P  Q3 = U Q4 = P  Q5 = U Q6 = U  Q7 = U | Q8 = U Q9 = U Q10 = U  Q11 = Y Q12 = Y  Q13 = U Q14 = U |
| 2023 Dingwall et al (22) | Feasibility and Acceptability of the Aboriginal and Islander Mental Health Initiative for Youth App: Nonrandomized Pilot With First Nations Young People | NT, regional | Mental health, mobile application | Culturally relevant  Culturally specific  Culturally adapted | No | Patients and community members | Community acceptance; Specific questions about cultural safety | C1 = 3 C2 = 3 C3 = 3 C4 = 3  C5 = 3 C6 = 3  C7 = 3 | | | C8 = 3 C9 = 3 C10 = 1  C11 = 3  C12 = 3  C13 = 2 | Q1 = P Q2 = P  Q3 = Y Q4 = Y  Q5 = U Q6 = U  Q7 = U | Q8 = P Q9 = U Q10 = U  Q11 = Y Q12 = Y  Q13 = Y Q14 = U |
| 2021 Freene et al (21) | An Aboriginal and Torres Strait Islander Cardiac Rehabilitation program delivered in a non-Indigenous health service (Yeddung Gauar): a mixed methods feasibility study. | ACT, metropolitan | Cardiovascular disease, cardiac rehabilitation program | Cultural safety  Cultural awareness  Culturally sensitive  Culturally informed | Yes | Patients / community members; Aboriginal and non-Indigenous professional | Community acceptance; Specific questions about cultural safety | C1 = 2 C2 = 3  C3 = 3 C4 = 3  C5 = 3 C6 = 3  C7 = 3 | | | C8 = 3 C9 = 3 C10 = 2  C11 = 2  C12 = 3  C13 = 3 | Q1 = U Q2 = P  Q3 = Y Q4 = P  Q5 = U Q6 = U  Q7 = U | Q8 = U Q9 = Y Q10 = Y  Q11 = Y Q12 = Y  Q13 = Y Q14 = Y |
| 2022 Guenther et al (23) | Suicide story: An evaluation of tackling suicide our way | NT, remote | Mental Health, Suicide prevention program | Cultural safety  Culturally specific  Culturally appropriate | Yes | Aboriginal health professional/ facilitator | Specific questions about cultural safety | C1 = 3 C2 = 3  C3 = 3 C4 = 2  C5 = 1 C6 = 0  C7 = 2 | | | C8 = 2 C9 = 0 C10 = 0  C11 = 2  C12 = 3  C13 = 2 | Q1 = U Q2 = P  Q3 = Y Q4 = Y  Q5 = U Q6 = U  Q7 = U | Q8 = U Q9 = Y Q10 = U  Q11 = Y Q12 = Y  Q13 = U Q14 = U |
| 2019 Ivers et al (37) | Home to health care to hospital: Evaluation of a cancer care team based in Australian Aboriginal primary care. | NSW, regional | Cancer, Cancer care program | Cultural safety  Culturally appropriate | Nil | Patients and community members | Specific questions about cultural safety | C1 = 1 C2 = 2  C3 = 2 C4 = 2  C5 = 1 C6 = 0  C7 = 2 | | | C8 = 1 C9 = 1 C10 = 0  C11 = 2  C12 = 0  C13 = 1 | Q1 = U Q2 = U  Q3 = U Q4 = U  Q5 = U Q6 = U  Q7 = U | Q8 = U Q9 = U Q10 = U  Q11 = Y Q12 = Y  Q13 = U Q14 = U |
| 2017 Lin et al (31) | Addressing disparities in low back pain care by developing culturally appropriate information for Aboriginal Australians: "My back on track, my future" | WA, rural | Low back pain, educational resource | Culturally appropriate  Cultural security  Culturally specific | Yes | Patients and community members | Community acceptance; Specific questions about cultural safety | C1 = 2 C2 = 2  C3 = 2 C4 = 2  C5 = 0 C6 = 3  C7 = 3 | | | C8 = 2 C9 = 0 C10 = 0  C11 = 2  C12 = 1  C13 = 3 | Q1 = U Q2 = P  Q3 = Y Q4 = Y  Q5 = U Q6 = U  Q7 = U | Q8 = U Q9 = U Q10 = U  Q11 = Y Q12 = Y  Q13 = U Q14 = U |
| 2022 Perdacher et al (34) | Using the Stay Strong App for the Well-being of Indigenous Australian Prisoners: Feasibility Study | QLD, mixed rurality | Mental health, mobile application | Culturally appropriate  Cultural safety | No | Patients and community members; Non-aboriginal health professional | Specific questions about cultural safety | C1 = 3 C2 = 3  C3 = 3 C4 = 3  C5 = 1 C6 = 0  C7 = 2 | | | C8 = 2 C9 = 2 C10 = 0  C11 = 2  C12 = 1  C13 = 3 | Q1 = U Q2 = U  Q3 = U Q4 = U  Q5 = U Q6 = U  Q7 = U | Q8 = U Q9 = U Q10 = U  Q11 = Y Q12 = Y  Q13 = U Q14 = U |
| 2016 Povey et al (29) | Acceptability of mental health apps for Aboriginal and Torres Strait Islander Australians: A qualitative study | NT, regional | Mental health, mobile applications | Culturally relevant  Culturally responsive  Culturally acceptable | No | Patients and community members | Community acceptance | C1 = 3 C2 = 3  C3 = 3 C4 = 2  C5 = 3 C6 = 1  C7 = 2 | | | C8 = 2 C9 = 3 C10 = 3  C11 = 3  C12 = 2  C13 = 2 | Q1 = U Q2 = U  Q3 = Y Q4 = Y  Q5 = U Q6 = U  Q7 = U | Q8 = U Q9 = U Q10 = U  Q11 = P Q12 = Y  Q13 = U Q14 = U |
| 2017 Quinn et al (30) | Enhancing the get healthy information and coaching service for Aboriginal adults: Evaluation of the process and impact of the program | NSW, mixed rurality | Chronic disease risk factors, Health coaching service | Culturally acceptable  Culturally appropriate  Culturally accessible | No | Patients and community members | Community acceptance | C1 = 2 C2 = 3  C3 = 3 C4 = 2  C5 = 0 C6 = 1  C7 = 2 | | | C8 = 3 C9 = 3 C10 = 1  C11 = 3  C12 = 2  C13 = 2 | Q1 = U Q2 = Y  Q3 = U Q4 = U  Q5 = U Q6 = U  Q7 = U | Q8 = U Q9 = U Q10 = U  Q11 = Y Q12 = Y  Q13 = U Q14 = U |
| 2010 Schoen et al (27) | Health promotion resources for Aboriginal people: lessons learned from consultation and evaluation of diabetes foot care resources. | WA, mixed rurality | Diabetes foot disease, educational resource | Culturally appropriate | No | Patients and community members; Aboriginal health professional/ facilitators | Community acceptance | C1 = 2 C2 = 3  C3 = 3 C4 = 2  C5 = 0 C6 = 3  C7 = 3 | | | C8 = 3 C9 = 1 C10 = 0  C11 = 2  C12 = 2  C13 = 0 | Q1 = U Q2 = P  Q3 = Y Q4 = Y  Q5 = U Q6 = U  Q7 = U | Q8 = U Q9 = U Q10 = U  Q11 = Y Q12 = Y  Q13 = U Q14 = U |
| 2019 Seear et al (38) | Piloting a culturally appropriate, localised diabetes prevention program for young Aboriginal people in a remote town. | WA, remote | Diabetes. Healthy lifestyle program | Cultural safety | No | Patients and community members | Community acceptance | C1 = 1 C2 = 1  C3 = 1 C4 = 3  C5 = 0 C6 = 1  C7 = 3 | | | C8 = 2 C9 = 1 C10 = 0  C11 = 3  C12 = 1  C13 = 2 | Q1 = P Q2 = P  Q3 = Y Q4 = Y  Q5 = P Q6 = U  Q7 = U | Q8 = U Q9 = U Q10 = U  Q11 = Y Q12 = Y  Q13 = U Q14 = U |
| 2016 Sinclair et al (24) | Positive community responses to an arts-health program designed to tackle diabetes and kidney disease in remote Aboriginal communities in Australia: a qualitative study. | WA, remote | Diabetes and kidney disease, Screening program partnered with Arts-based health education | Culturally appropriate | No | Patients and community members | Community acceptance | C1 = 2 C2 = 2  C3 = 2 C4 = 2  C5 = 3 C6 = 1  C7 = 2 | | | C8 = 2 C9 = 2 C10 = 2  C11 = 2  C12 = 1  C13 = 2 | Q1 = U Q2 = P  Q3 = Y Q4 = P  Q5 = U Q6 = U  Q7 = U | Q8 = U Q9 = P Q10 = U  Q11 = Y Q12 = Y  Q13 = P Q14 = U |
| 2012 Taylor et al (28) | Intercultural communications in remote Aboriginal Australian communities: What works in dementia education and management? | NT, remote | Dementia, educational program | Cultural safety  Culturally appropriate | Yes | Patients and community members; Aboriginal and non-Indigenous professional | Community acceptance | C1 = 3 C2 = 2  C3 = 2 C4 = 2  C5 = 0 C6 = 0  C7 = 2 | | | C8 = 1 C9 = 0 C10 = 0  C11 = 2  C12 = 1  C13 = 2 | Q1 = U Q2 = P  Q3 = Y Q4 = P  Q5 = U Q6 = U  Q7 = U | Q8 = U Q9 = U Q10 = U  Q11 = P Q12 = Y  Q13 = U Q14 = U |
| 2020 Tighe et al (32) | Usage and acceptability of the iBobbly app: Pilot trial for suicide prevention in aboriginal and torres strait islander youth | NT, remote | Mental health, mobile application | Culturally safe  Culturally appropriate | No | Patients and community members | Community acceptance; Specific questions about cultural safety | C1 = 2 C2 = 3  C3 = 3 C4 = 2  C5 = 1 C6 = 1  C7 = 2 | | | C8 = 2 C9 = 2 C10 = 1  C11 = 2  C12 = 0  C13 = 1 | Q1 = U Q2 = U  Q3 = Y Q4 = U  Q5 = U Q6 = U  Q7 = U | Q8 = U Q9 = U Q10 = U  Q11 = Y Q12 = Y  Q13 = U Q14 = U |
| 2014 Truasheim et al (39) | Cultural Safety for Aboriginal and Torres Strait Islander Adults within Australian Music Therapy Practices. | QLD, metropolitan | Any chronic disease, Music therapy program | Cultural safety | Yes | Patients and community members | Community acceptance | C1 = 3 C2 = 2  C3 = 2 C4 = 1  C5 = 0 C6 = 0  C7 = 2 | | | C8 = 1 C9 = 0 C10 = 0  C11 = 2  C12 = 0  C13 = 0 | Q1 = P Q2 = U  Q3 = U Q4 = U  Q5 = U Q6 = U  Q7 = U | Q8 = U Q9 = U Q10 = U  Q11 = P Q12 = Y  Q13 = U Q14 = U |
| 2022 West et al (33) | Yarning about foot care: evaluation of a foot care service for Aboriginal and Torres Strait Islander Peoples | NSW, metropolitan | Diabetic foot disease, Foot care service | Cultural safety  Culturally appropriate  Cultural capability  Cultural awareness | Yes | Patients and community members | Community acceptance | C1 = 2 C2 = 3  C3 = 3 C4 = 3  C5 = 1 C6 = 2  C7 = 3 | | | C8 = 2 C9 = 1 C10 = 0  C11 = 3  C12 = 0  C13 = 2 | Q1 = P Q2 = U  Q3 = Y Q4 = U  Q5 = U Q6 = U  Q7 = U | Q8 = U Q9 = Y Q10 = U  Q11 = Y Q12 = Y  Q13 = U Q14 = U |

***** Location of intervention reported by Australian state (QLD = Queensland, NSW = New South Wales, VIC = Victoria, NT = Northern Territory, WA = Western Australia, SA = South Australia, ACT = Australian Capital Territory). The Modified Monash scale has been used to indicate rurality (Department of Health and Aged Care. Modified Monash Model Canberra: Australian Government; 2023 [updated 12th December 2023. Available from: <https://www.health.gov.au/topics/rural-health-workforce/classifications/mmm>)

**†** The provision of any definition for any term related to cultural safety (or similar) in the paper is coded as Yes

**‡** Quality assessment with diverse studies (QuADS) score rated from 0 (no mention) to 3 (detailed exploration of criteria within manuscript). Criterion 1 (C1) = conceptual underpinning, C2 = research aims, C3 = research setting, C4 = study design, C5 = sampling, C6 = rational for data collection tools, C7 = appropriate data collection tool, C8 = data collection description, C9 = recruitment, C10 = analysis justification, C11 = appropriate analysis method, C12 = stakeholder involvement, C13 = strengths / limitations

^Aboriginal and Torres Strait Islander quality appraisal tool questions answered as Yes, No, Partially or unclear. Q1 = community priority, Q2 = community engagement, Q3 = Aboriginal and Torres Strait Islander leadership, Q4 = Aboriginal and Torres Strait Islander governance, Q5 = community protocols followed, Q6 = Agreements related to existing community IP, Q7 = agreements related to new community IP, Q8 = community control over data collection and management, Q9 = Indigenous research paradigm, Q10 = strengths-based approach, Q11 = findings translated into policy / practice, Q12 = Community benefit, Q13 = Mutual learning opportunities
